# Racism in Australia: a protocol for a systematic review and meta-analysis

**DOI:** 10.1186/s13643-022-01919-2

**Published:** 2022-03-17

**Authors:** Jehonathan Ben, Amanuel Elias, Ayuba Issaka, Mandy Truong, Kevin Dunn, Rachel Sharples, Craig McGarty, Jessica Walton, Fethi Mansouri, Nida Denson, Yin Paradies

**Affiliations:** 1 Centre for Resilient and Inclusive Societies (CRIS), Melbourne, Victoria, Australia; 2 Alfred Deakin Institute for Citizenship and Globalization, Faculty of Arts and Education, Deakin University, Melbourne, Victoria Australia; 3 School of Health and Social Development, Deakin University, Melbourne, Victoria Australia; 4 School of Nursing and Midwifery, Monash University, Melbourne, Victoria Australia; 5 Menzies School of Health Research, Darwin, NT Australia; 6 School of Social Sciences, Western Sydney University, Penrith, New South Wales Australia; 7 School of Psychology, Western Sydney University, Penrith, New South Wales Australia

**Keywords:** Racism, Racial discrimination, Australia, Prevalence, Systematic review, Meta-analysis, Health outcomes

## Abstract

**Background:**

Racism has been identified as a major source of injustice and a health burden in Australia and across the world. Despite the surge in Australian quantitative research on the topic, and the increasing recognition of the prevalence and impact of racism in Australian society, the collective evidence base has yet to be comprehensively reviewed or meta-analysed. This protocol describes the first systematic review and meta-analysis of racism in Australia at the national level, focussing on quantitative studies. The current study will considerably improve our understanding of racism, including its manifestations and fluctuation over time, variation across settings and between groups, and associations with health and socio-economic outcomes.

**Methods:**

The research will consist of a systematic literature review and meta-analysis. Searches for relevant studies will focus on the social and health science databases CINAHL, PsycINFO, PubMed and Scopus. Two reviewers will independently screen eligible papers for inclusion and extract data from included studies. Studies will be included in the review and meta-analysis where they meet the following criteria: (1) report quantitative empirical research on self-reported racism in Australia, (2) report data on the prevalence of racism, or its association with health (e.g. mental health, physical health, health behaviours) or socio-economic outcomes (e.g. education, employment, income), and (3) report Australian data. Measures of racism will focus on study participants’ self-reports, with a separate analysis dedicated to researcher-reported measures, such as segregation and differential outcomes across racial/ethnic groups. Measures of health and socio-economic outcomes will include both self-reports and researcher-reported measures, such as physiological measurements. Existing reviews will be manually searched for additional studies. Study characteristics will be summarised, and a meta-analysis of the prevalence of racism and its associations will be conducted using random effects models and mean weighted effect sizes. Moderation and subgroup analyses will be conducted as well. All analyses will use the software CMA 3.0.

**Discussion:**

This study will provide a novel and comprehensive synthesis of the quantitative evidence base on racism in Australia. It will answer questions about the fluctuation of racism over time, its variation across settings and groups, and its relationship with health and socio-economic outcomes. Findings will be discussed in relation to broader debates in this growing field of research and will be widely disseminated to inform anti-racism research, action and policy nationally.

**Systematic review registration:**

PROSPERO CRD42021265115.

**Supplementary Information:**

The online version contains supplementary material available at 10.1186/s13643-022-01919-2.

## Background

### Introduction

Racism is an enduring structural phenomenon that detrimentally impacts the social fabric of societies, human relations and community wellbeing across time [1, 2]. While debates remain regarding what constitutes racism, in this protocol, we define racism as a historical and ongoing system of oppression, which creates hierarchies between social groups based on perceived differences relating to origin and cultural background [1, 3]. These hierarchies disadvantage some groups and advantage others, generating and exacerbating unfair and avoidable inequalities [4]. Racism is multi-faceted and is manifest in structural, institutional, interpersonal and internalised forms. It is expressed and reinforced through policies, practices, media representations, stereotypes, prejudice and discrimination. Racism draws on characteristics such as ‘race’, ethnicity, nationality and religion and is related to constructs such as Islamophobia, anti-Semitism and xenophobia [5].

Racism has recently drawn more attention worldwide, whether in relation to increased protest against institutional discrimination and social and health inequities affecting Black and Indigenous peoples or xenophobic sentiments under COVID-19. In Australia, as in other parts of the world, racism has been entwined with European colonialism and emerged from the use of race as a system of rule (e.g. [6, 7]). Racism in Australia has endured since British colonisation in 1788 and has derived initially from the relations of extraction, exploitation, expropriation and competition between British colonisers and Indigenous peoples (e.g. [8]), which were later extended to discriminate against and exclude different immigrant populations. In relation to the past decade or so, racism in Australia has been directed in different ways towards numerous ethnic, racial, national, religious and migrant groups, with each racialised differently under colonial rule and targeted by various forms of racism. These have included (but have not been limited to) violence against South Asian students, inflammatory rhetoric and inhumane policies towards asylum seekers, Islamophobia and racist incidents targeting Muslim Australians, mediatised racialisation and episodic criminalisation of African Australians, the spread of race-based hatred online, attacks against Asian Australians in the context of COVID-19 and the Australian government’s use of heavy border restrictions to police and contain those people whom it deems undesirable (e.g. [9–13]). Meanwhile, colonisation and profound structural racism towards Indigenous peoples﻿ persist, manifesting in all areas of life, including intergenerational traumas, non-recognition of Indigenous rights, health inequalities, poverty and poor education, over-incarceration and deaths in custody [14].

While racism remains among the most profound social and public health issues of our times, recognition of its ill effects and new anti-racism initiatives are growing too. Racism research continues to evolve and accumulate, which parallels these developments (see recent reviews in [15, 16]). This includes many empirical, quantitative studies that are often based on surveys of participants’ self-reports and gather data about both experiences and expressions of racism [15]. Despite the accumulation of such studies over at least four decades, this body of research has yet to be comprehensively reviewed and collectively analysed within a single study focused on racism in Australia. This means that important questions discussed in this literature have yet to be considered, including, for instance, about how the prevalence of racism may change over time, the extent to which it may vary depending on racism’s diverse forms and settings, and how different racial, ethnic and other social groups experience and/or perpetrate it. Questions examining the nuances in racism’s prevalence disaggregated by groups, cohorts and time periods (duration and spells), need further considerations, as they are critical for better understanding of racism’s intersectional and longitudinal impacts. Likewise, the impact of racism on health and its connection to various socio-economic phenomena such as education and employment have yet to be jointly synthesised and discussed within the Australian context.

Australia is unique in its cultural, linguistic and religious diversity. Aboriginal and Torres Strait Islander peoples, the Indigenous peoples of Australia, represent 3.3% of Australia’s population and comprise over 250 Australian Indigenous language groups [17, 18]. Almost half (45% or 10.6 million) of the population were either born overseas or have one or both parents who were born overseas [19], a higher proportion than the USA, Canada and Britain. Twenty-one percent of Australians speak a language other than English at home and increasing proportions of migrants arriving in Australia are coming from China and India. Furthermore, greater than two thirds of all Australians report religious affiliation. The most common religious affiliation reported is Christianity (52%), followed by Islam (2.6%) and Buddhism (2.4%). Australia’s religious diversity is increasing, with the proportion who report a religious affiliation other than Christian growing from 5.6% in 2006 to 8.2% in 2016. This was spread across most non-Christian religions, with Islam (1.7% to 2.6%) and Hinduism (0.7 to 1.9%) showing the highest increase [20].

The rest of this protocol is organised as follows. The next sub-sections discuss previous Australian racism research, and the rationale, aims and key hypotheses guiding our study. The ‘Methods and design’ section outlines the review methods and protocol that will be followed. The ‘Data analysis and critical appraisal’ section describes the analytical and critical appraisal strategy that will be used, while the study’s limitations and dissemination strategy are discussed in the protocol’s final section.

### Previous studies

Previous quantitative research in Australia has provided considerable attention to identifying racism’s nature and measuring its prevalence and impact. Various empirical, survey-based studies have examined racism and related phenomena using different conceptualisations and research questions, focussing also on phenomena such as discrimination, prejudice, Islamophobia and anti-immigrant sentiments. Their differential concepts, wide-ranging measures and diverse study designs, likely affect the prevalence rates they report. For example, the Scanlon Foundation’s nationally representative Mapping Social Cohesion (MSC) survey found that experiences of discrimination based on skin colour, ethnicity or religion have ranged between 9 and 20% in 2007–2020 [21], while other studies have usually reported higher rates. In the 2014 national General Social Survey (GSS), 34% of respondents reported experiencing racial/ethnic discrimination [22], whereas the 2015–2016 national Face Up to Racism survey found that forms of everyday racism such as name-calling, mistrust and disrespect were reported by 34–40% of participants [23]. However, in response to questions about racism more generally, respondents have acknowledged that the prevalence of racism is much higher. In Face Up to Racism, 79% agreed that racial prejudice exists in Australia in general, though only 11% self-identified as racist [24], while 86% in the national Geographies of Racism survey agreed with this proposition [25]. Similar findings about individuals as perceiving more discrimination towards their group than personally have been discussed elsewhere (e.g. [26]).

Various studies have focused on experiences of racism across particular social groups. They have discussed the variable circumstances, contexts and arrangements that shape forms and experiences of racism and that appear to affect variabilities in the prevalence and impact of racism across groups. Face Up to Racism, for instance, found that concerns about one’s closest relative marrying a person from different racial, national and other out-groups varied tremendously across nine groups and peaked at 36–63% for Indigenous, African, Jewish and Muslim out-group members [23]. Variations can also be found among studies focused on specific groups, for instance, Indigenous peoples, who have been among the most widely studied in racism research [15]. National studies of mixed age groups using repeated cross-sectional designs have found different rates of unfair treatment of Aboriginal or Torres Strait Islanders, including 15% in the 2012–2013 National Aboriginal and Torres Strait Islander Health Survey (NATSIHS) [27], 35% in the 2014–2015 National Aboriginal and Torres Strait Islander Social Survey (NATSISS) [28], and 37% in the 2018 Mayi Kuwayu Study ([29], p. 5).

Another group of surveys has measured different racist attitudes against immigrants. For example, across Australian Values Study (AVS) data collected between 1981 and 2019, between 5 and 11% of participants indicated dislike of having immigrants or foreign workers as neighbours [30], whereas 29% of participants in a study focussed on Melbourne said they would be reluctant to move into a neighbourhood where many new migrants are living [31]. In November 2020, Markus ([21], p. 76) found that 18% of respondents agreed that it should be possible to reject immigrants on the basis of their race or ethnicity and that 24% agreed that rejection should be possible due to religion. Finding about migrants’ experiences vary as well. The Longitudinal Study of Immigrants in Australia (LSIA) found in the mid-2000s that over 40% of respondents thought racism existed in Australian society ([32], p. 3), while more recently, another study found that experiences ranged considerably, from 2 to 32% among eight different groups (e.g. Caucasian Australians, Indian, Chinese, Arabic-speaking) [33]. Anti-asylum seeker sentiment has also been measured across a number of surveys. The Face Up to Racism report found that 43% agreed that asylum seeker boats should be turned back [23]. The 2019 MSC found that 47–50% of the population were ‘not concerned’ that Australia is too harsh in its treatment of asylum seekers and refugees ([34], p. 65).

Recent racism research has also given considerable attention to Muslim Australians. A national survey showed that 20% of non-Muslim participants stated that they would rather not live in a place ‘where there are Muslims’ [35], while another study found unfavourable opinions about Muslims and about Islam among 12% and 27% of participants, respectively [36]. In other surveys, as many as 63% and 56% of participants were concerned about marrying someone of a Muslim faith [23, 37].

Children and young people are another group who are increasingly focused on in recent research. One longitudinal study found differences in the prevalence of racial discrimination as experienced by children aged 10–11 of various groups, including among children of Anglo/European background (8%), visible minority background (18%) and Indigenous background (25%), with a reduction in these rates occurring in ages 14–15 [38]. Meanwhile, in another study, caregivers reported racial discrimination (conceived as being bullied or treated unfairly due to being Aboriginal) by 20% of Indigenous children aged 4–11 at school [39]. Other research, on school students in years 5–9 from Victoria and New South Wales, showed that 50% of Indigenous students experienced direct discrimination. Rates were lower for young people from European (38%) and Anglo (25%) backgrounds and higher for various migrant groups (58–67%) [40].

Studies also suggest that experiences of racism vary across settings. Face Up to Racism, for example, found that experiences were more common in settings such as public transport/on the street, workplaces, education and shops/restaurants, all of which were reported by over 30% of respondents. This finding is consistent with other studies, for example from the state of Victoria, where these same settings were noted as most common, with the prevalence of racism ranging between 23 and 35% [41]. However, other studies suggest rates may be higher in schools and neighbourhoods and may vary greatly across exposure types (e.g. direct, vicarious) (e.g. [31, 40]). Meanwhile, a national study focussed on online settings demonstrates the potential gap between direct and indirect experiences in these settings﻿; while only 5% were personally targeted, 35% witnessed cyber racism ([42], p. 72).

Racism research in Australia has also examined its impact, with an important focus given especially to health. Multiple studies show that racism is negatively associated with depression and distress/worry (e.g. [43–45]) and with emotional difficulties and wellbeing (e.g. [40, 46, 47] and see subgroup analysis among Australian adolescents in [48]). For physical health, results are more mixed. For instance, in a study of Indigenous children, exposure to racial discrimination was associated with sleep difficulties and obesity, but not with being underweight [39], while among Australian children, exposure to racism was associated with an increased risk of being overweight or obese and with higher body mass index (BMI) measures [38], and experiences of religious discrimination were associated with socioemotional adjustment and sleep outcomes [49]. Divergence between the results for mental and physical health may be in line with international analysis (e.g. [50, 51]), yet this remains to be tested.

Finally, associations between racism and socio-economic status indicators, such as education, employment and income, may be complex. For example, research conducted internationally found that racism may inhibit access to employment (e.g. [52, 53]) or negatively affect academic performance and achievement [47, 48], impose significant health economic cost [54] and reduce workplace productivity [55]. Education may also play a key role in awareness regarding racism. Some studies have shown that a higher level of education is associated with increased awareness and perception of racism and that education fosters more adherence towards structural than individualist explanations of racial inequality [56, 57], thus propelling us to further consider racism in relation to multiple possible causes and effects.

### Rationale

Despite ongoing concerns about racism in Australia, and a recent surge in quantitative research that find racism to be pervasive and harmful, the cumulative evidence base on the prevalence and effects of racism has yet to be systematically reviewed or meta-analysed. To date, the majority of racism research has focussed on the USA, as indicated by reviews and meta-analyses of this scholarship. These studies’ findings may not be applicable to the Australian context given numerous cross-country differences, from immigration history to present day demographics. A few small-scale, group-specific reviews and meta-analyses have been conducted that centre specifically on Australia. One meta-analysis found associations between racism and academic and socio-emotional wellbeing outcomes among Australian adolescents, which, for academic outcomes, were stronger than for the USA [48, 50]. Another systematic review found links between racism and negative schooling experiences among Indigenous peoples [47]. An additional study is currently underway, which will focus on the impact of racism on mental and physical health among Indigenous peoples as well [58]. Meanwhile, individual national empirical studies using primary data are, inevitably, limited to specific research questions and study designs and focus on certain measures of racism. For example, the MSC, conducted annually since 2007, provides extensive data on experiences over time and across states and subgroups. However, it focuses on a general measure of discrimination based on skin colour, ethnic origin or religion, which may underestimate the extent of discrimination compared with questions about specific locations ([21], p. 87), is rarely reported in peer-reviewed articles, and does not examine racism’s associations with phenomena such as health.

There remain other unanswered questions about racism. As discussed in the previous sections, the prevalence of racism ranges widely between studies, based on different study designs, participant characteristics and the adopted measures of racism. Furthermore, while racism in Australia operates mostly subtly (e.g. [59, 60]), the experience of many people, especially Asian migrants, during the COVID-19 pandemic, may suggest that blatant forms of racism are alive and well, while increased awareness is nowadays given to vicarious, structural and online racism [61, 62]. Structural racism, which has received increasing coverage in the literature, particularly in USA [63], remains to be systematically studied in Australia. Emerging studies on Indigenous deaths in custody, and on housing, healthcare and workplaces, indicate that racism occurring at the structural level can significantly impact the wellbeing of Indigenous peoples and migrants [7, 64–67]. What is more, while there are indications that racism is more commonly experienced in some settings (e.g. workplaces, schools, media, sport, shops and healthcare), its prevalence varies across settings [25, 68, 69]. Understanding such patterns of reporting racism, including instances where racism occurs but remains unreported or underreported, may have serious implications for anti-racism agendas.

Another research gap concerns the fluctuation of racism over time, which may reflect changing social, economic and political processes and events, as well as changes to how racism is conceptualised or to how it is understood over time (e.g. [47]). Different racial, ethnic, national and religious groups may be subject to variable exposures to and effects of racism, but some groups have been less considered (e.g. various religious groups), while others have been more widely studied, but evidence from individual studies has yet to be synthesised (e.g. Muslim Australians, Asian Australians). Statistical comparisons across racial, ethnic, national and religious groups are uncommon in Australia as well. Intersections between these and other demographics such as age and socio-economic status are being increasingly examined, especially in health research [28, 70], and may now be possible to meta-analyse.

### Aims

This project will provide a timely and critical synthesis of existing quantitative data to enhance our understanding of racism in Australia. The study has three principal aims: (1) to examine the key characteristics of quantitative studies on self-reported racism in Australia. This will include study locations, periods, designs, sampling techniques and sample sizes; study participants’ age, sex and racial/ethnic/national backgrounds; racism measures used, including instruments, timeframes, number of items, types (e.g. interpersonal, vicarious, structural) and forms (e.g. blatant/subtle; physical, verbal, mistrust, disrespect) of racism. We will also discuss study types and data that are currently lacking; (2) to examine the prevalence of racism, as experienced and expressed by study participants, across different types and forms, over time periods, across states and in various settings and institutions (e.g. work, education, public, policing and justice), and between groups (e.g. racial, ethnic, national, religious). Measures of structural and systemic racism that are not based on study participants’ self-reports, such as researcher-reported ecological measures of segregation and differential outcomes in settings such as the labour market or the justice system, will be reviewed separately and synthesised descriptively; (3) to examine the direction and magnitude of associations between reported racism and key health outcomes, including negative mental health, positive mental health, physical health and general health and between reported racism and socio-economic outcomes, including education, employment status and income.

### Hypotheses

Based on the aforementioned findings from previous individual studies conducted in Australia, and on international reviews and meta-analyses, we propose the following hypotheses: (1) the prevalence of racism will vary considerably depending on study designs, locations and measures of racism; (2) reported experiences of racism will become more prevalent over time; (3) ‘subtle’ forms of racism (e.g. exclusion, mistrust) will be more prevalent than ‘blatant’ forms (e.g. physical attack, slurs) and reports of structural and systemic racism more prevalent than reports of interpersonal racism; (4) racism will be more prevalent in settings that relate to work, education and public spaces, and the prevalence of racism online will rise most significantly over time, especially since COVID-19; (5) Indigenous peoples and migrants from various minority backgrounds will report more racism than white/European Australians, and experiences of racism will be most prevalent among Indigenous peoples and Muslim Australians; (6) racism will have the strongest associations with mental health outcomes and weaker associations with general and physical health outcomes; and (7) racism will be associated with higher education level, employment and income.

## Methods and design

### Design

The research will consist of a systematic literature review and meta-analysis, following the Preferred Reporting Items for Systematic reviews and Meta-Analyses (PRISMA) guidelines and criteria [71].

### Criteria for considering studies

#### Type of studies

We will include empirical studies that examine racism’s prevalence and/or associations with health and socio-economic outcomes in Australia. The review and meta-analysis will focus on survey-based studies reporting quantitative data. Empirical data will be collated from a variety of sources, including past and ongoing datasets, national surveys and other survey-based quantitative studies. While our focus will be primarily on published studies (including articles, books and book chapters), we will also include reports that have been published and copyrighted, where the data reported in them have not been otherwise discussed in peer reviewed publications. Dissertations, conference papers, presentations and public opinion polls will be excluded. All study populations will be included, regardless of participants’ demographics.

#### Exposure measures

This study will focus on measures of exposure to racism, based on study participants’ self-reports. We focus on participants’ self-reports, and particularly on survey-based designs, as a central way of learning about racism directly from people who may experience and perpetrate it, and as measures that are widely used in quantitative studies of racism, which would make them amenable to meta-analysis. We will use a broad definition of racism, in line with the definition in the ‘Introduction’ section, to account for differential conceptualisations across studies. Both experiences and expressions of racism by study participants will be included. The study will include reports of interpersonal exposure, vicarious reports (e.g. witnessing or knowledge of racism as experienced by someone else) and reports about structural racism (e.g. as existing in Australia or generally in certain settings or towards specific groups). Self-reported expressions of racism will include measures of attitudes, beliefs and behaviours. Researcher-reported measures of structural and systemic racism (e.g. racial segregation, differential outcomes among racial/ethnic groups in the labour market, housing market and in relation to the justice system) will be reviewed separately from studies using participants’ self-reports, and descriptively synthesised, but since they rely on different measurement types, they will not be meta-analysed. All timeframes of exposure to racism will be included. Measures of discrimination or prejudice in general and forms that do not meet our definition of racism (e.g. discrimination based on gender, age) will be excluded. Measures of researcher-reported racism such as experimental exposures (e.g. videos, vignettes, tasks) will be excluded as well.

#### Prevalence measures

All measures of the prevalence of racism will be included, including its different forms, time periods, settings and affected groups.

#### Outcome measures and covariates

A growing amount of data is available on the relationship between racism and other phenomena. In this study, we are especially interested in key health and socio-economic outcomes, whose links with racism have been relatively widely discussed and measured. The following health outcomes will be included: (1) negative mental health (e.g. depression, psychological distress, stress, anxiety, social and emotional difficulties); (2) positive mental health (e.g. self-esteem, life satisfaction, control, well-being); (3) physical health (e.g. high blood pressure, obesity); (4) general health (e.g. feeling unhealthy, sleep, combined measures of physical and mental health); and (5) health behaviours (e.g. alcohol, tobacco or substance use). We will also include measures of the following socio-economic outcomes among study participants, where studied as covariates alongside racism measures: (6) education (e.g. highest qualification, level completed); (7) employment (e.g. labour force status); and (8) income/finances (e.g. income scales). We will examine these as proxies of class and will consider their intersection with race and the prevalence and effects of racism. Measures of health and socio-economic outcomes will include both self-reports and researcher-reported measures (e.g. cortisol for stress, BMI for obesity).

### Identification of eligible studies and data extraction

#### Search strategy

Our searches will cover the following major online, social and health science databases: CINAHL, PsycINFO, PubMed and Scopus. Searches will only include materials published in English. They will not specify an earliest search date and will run to the present. For a list of search terms that will be used, please see Additional file 1. Existing reviews and meta-analyses will be manually searched for other relevant studies.

#### Selection of studies

All search results will be imported into the software Covidence [72]. Once duplicates are deleted, two reviewers will independently screen all titles and abstracts to assess eligibility for inclusion. The full texts of potentially eligible studies will be obtained and screened to confirm they meet the following criteria: (1) report empirical research and quantitative data, (2) report racism’s prevalence or its association with socio-economic outcomes (e.g. education, employment, income) or health outcomes (e.g. mental health, physical health, health behaviours, general health) and (3) report Australian data. Any discrepancies between reviewers during the screening process will be resolved by consensus, and further disagreement will be resolved by a third reviewer.

#### Data extraction

Two independent reviewers will review and extract data from each paper using Covidence. Disagreements will be resolved by consensus between the reviewers or by a third reviewer. Where the same study appears in multiple publications, we will retain as much data as possible without ‘double-counting’ such studies. Each study will be reported once. Data will be extracted in five different categories or levels, namely (1) study data, (2) participant data, (3) racism exposure data, (4) prevalence data and (5) association data. We provide details for each level next.

(1) Study level data will include the study’s full reference, type of publication (e.g. book chapter, journal article), data source name (e.g. GSS, LSIA), study location (e.g. state and/or city or other location, or nationally), study setting (e.g. healthcare, schools), year/s of racism data collection, data type (e.g. longitudinal, cross-sectional) and sampling procedure (representative or non-representative). (2) Participant data will include the overall sample size, participant age range, participant sex, and participant racial, ethnic, and/or national background. (3) Racism exposure data will include the instrument name, type of racism measured, settings (e.g. workplaces, healthcare) and the timeframe of exposure to racism (e.g. ‘over the past 12 months’). (4) Prevalence data will include data such as event rates and sample sizes, events and non-events, and means, standard deviations and sample sizes. Where the same study reports data separately for different exposure measures (e.g. racism of different types, racism experienced in different settings), exposure timepoints (e.g. baseline/post 12 months exposure to racism) and subgroups who experience or express racism (e.g. males/females, Indigenous/non-Indigenous), data for each measure will be recorded separately, and the specific exposure measure, timepoint and subgroup will be noted. (5) Association data will include data on the associations between racism and health and socio-economic outcomes, including the exposure name, outcome name, timepoint when the association was measured, subgroup, and whether the association is unadjusted or adjusted for other variables. For adjusted associations, we will note the names of all other variables included in the most elaborate multivariate model. We will extract sample sizes, test results and the types of data reported, including, for example, correlations; odds ratios (ORs) and confidence intervals (CIs); counts of events and non-event; means and standard deviations; and standardised mean differences (Cohen’s *d*).

## Data analysis and critical appraisal

### Analysis

The characteristics of studies that meet our inclusion criteria will be summarised, and studies using self-reported measures and relevant metrics (e.g. prevalence data, association data) will be meta-analysed using Comprehensive Meta-Analysis (CMA) Version 3.0 [73]. We anticipate that racism prevalence data will be mainly reported as event rates (i.e. percentages) and sample sizes, and therefore, rates will be the final metric used in the analysis of prevalence. Where possible, other prevalence metrics will be converted into rates. For the analysis of associations between racism, health and socio-economic outcomes, we anticipate that correlation coefficients and sample sizes will be the most widely used metric across studies, and we will therefore use them as our final metric. Where possible, other metrics will be converted into correlations, including, for example, odds ratios (ORs) and confidence intervals (CIs); means, standard deviations and sample sizes for two groups (racism and no racism); and standardised mean differences (Cohen’s *d*) and sample sizes for two groups. Studies that do not report appropriate and sufficient association data will be reported in the review but excluded from the meta-analysis.

We anticipate that the main analysis will use random effects models and mean-weighted effect sizes. In line with previous meta-analyses on racism and health (e.g. [51, 74]), we anticipate that random effects models will be more appropriate for aggregating effect sizes than fixed effects models, because of the various differences expected across studies, and since we aim to generalise findings to the population of studies reporting quantitative data on racism in Australia. Weighted effect sizes will be calculated using CMA, to account for variation in sample sizes, thus giving more weight to effects from larger samples. We will assess the heterogeneity of effect sizes among studies that focus on similar health outcomes or socio-economic covariates by using the *Q* and *I*^*2*^ statistics. Moderation analyses will be used to further explain heterogeneity between studies. Using mixed effect models, we will compare racism’s prevalence and associations over time and across exposure measures, forms and settings. Subgroup analyses will be conducted to examine prevalence and associations based on ethnic, racial, national and religious backgrounds, as well as age and sex. Should enough studies be available, the review will also consider subgroup analysis comparing the prevalence of racism in Australia before and during the COVID-19 pandemic, especially against people from Asian backgrounds.

The prevalence and effects of researcher-reported measures of structural and systemic racism will be synthesised descriptively. Vote counting will be used to synthesise findings of multivariate models for health and socio-economic outcomes. Such models would likely consist of diverse sets of variables that would pose challenges to meta-analytic methods such as meta-regression which require a standard set of covariates.

### Publication bias

Three methods will be used to assess publication bias (that is, the possibility that significant results may be more likely to be published): (1) Egger’s weighted regression method will be used, and we will examine the regression intercept’s significance for statistical evidence of bias; (2) Failsafe *N* will be calculated to estimate how many additional, unlocated studies (with an average effect size of zero) would be required to change a significant result to a non-significant one [75]. Where we find publication bias, Duval and Tweedie’s trim-and-fill method will be used which adjusts for unreported, missing studies [76, 77]; (3) We will also assess the symmetry of funnel plots of study prevalence and effects as visual evidence of bias. These methods will draw on tests and visualisations available on CMA.

### Critical appraisal

Joanna Briggs Institute (JBI) critical appraisal tools [78] will be used to determine study quality, accounting for different study designs**.** We will also gauge study quality by conducting moderation analyses of study quality indicators. We will compare racism’s prevalence and associations between publication types (published versus unpublished), study designs (representative versus non-representative), study sampling (random versus non-random), sample sizes (larger samples versus smaller samples) and characteristics of exposure measures, such as instrument type and number of items.

## Discussion

### Strengths and limitations of the review

A major strength of this study is its novelty, as the first comprehensive overview of the prevalence and effects of racism in Australia. While findings on racism as pervasive and harmful are commonplace, for the first time, we will be able to look across this evidence base, and quantify racism’s prevalence and effects cumulatively. Our comprehensive approach will allow us to draw wider conclusions beyond current reviews and beyond individual studies focused on national samples. We will be able to summarise and synthesise findings rigorously and to characterise this growing field and its current state of affairs to date. Methodologically, this study will draw on robust, established practice in systematic reviews and meta-analysis. We will build on previous scholarship by members of our team, such as reviews and meta-analyses on racism globally [51, 74, 79], and analyses and reviews on racism in Australia (e.g. [10, 37]). Another strength of this study is its national scope. While racism remains a strongly interconnected global phenomenon, given the present consolidation of nationalisms under COVID-19, and the recent increase in Australian studies, a national-level investigation may be particularly useful to researchers, community groups, activists, policymakers and others working to combat racism in Australia.

One limitation of this review is its focus on participants’ self-reports. While we have explained the reasons for this focus earlier, we are aware that self-reports may be highly variable, can be shaped by response biases and may be at odds with how racism operates and manifests more broadly (e.g. institutionally, structurally). For example, despite evidence about the existence of racism, participants may deny, downplay and under-report expressing or experiencing it (e.g. [80, 81]). By reviewing and synthesising researcher-reports in addition to self-reports, the study will provide a broader picture of the prevalence and impacts of racism. Possible discrepancies in findings across study designs and types of reporting will be discussed, and we will further analyse them where feasible methodologically. Another possible limitation to this review may be that some publications of racism data are available only as ‘grey literature’ (e.g. research reports) and may not meet standards for peer-reviewed research. Our current work in this field suggests that this literature is sizable [15] and that various important data are not discussed outside of such reports. We will consider this possibility through assessment of study quality and publication bias, although we may be limited due, for example, to reports’ minimal discussions of study methodologies. Another limitation may be heterogeneity in conceptualisation and measurement of racism across studies, with studies reporting results that may not be readily summarised in a meta-analysis. In addition, while adequate data may be available on some forms and settings of racism, and for some subgroups, it may be more limited for others, which may inhibit certain analyses. Finally, we acknowledge that the review will provide only a partial picture of racism research in Australia since it will not cover the substantial qualitative scholarship on the topic. A review of qualitative racism research would be able to illuminate additional questions that cannot be addressed in the current review and remains an important undertaking for future research.

### Dissemination

This study will provide a novel and comprehensive synthesis of the quantitative evidence base on racism in Australia. Findings will be widely disseminated to inform anti-racism research, action and policy nationally. The review will also inform the fight against racism in other countries, particularly countries in the Global North shaped by settler colonialism and intensive migration (e.g. Canada, New Zealand, USA). Researchers in other countries may replicate this study at the national level. The study will be accessible to academics and the wider public through publication in an open-access peer-reviewed journal. A summary of the results will be made available also through factsheets and potentially discussed in a Centre for Resilient and Inclusive Societies (CRIS) policy paper. Findings will be further disseminated through CRIS and university communications channels, as well as through conference presentations.

## Supplementary Information

**Additional file 1.** Racism in Australia.

## Data Availability

Not applicable.

## Abbreviations

AVS: Australian Values Study
BMI: Body Mass Index
CI: Confidence interval
CMA: Comprehensive Meta-Analysis
CRIS: Centre for Resilient and Inclusive Societies
GSS: General Social Survey
JBI: Joanna Briggs Institute
LSIA: Longitudinal Study of Immigrants in Australia
MSC: Mapping Social Cohesion survey
NATSIHS: National Aboriginal and Torres Strait Islander Health Survey
NATSISS: National Aboriginal and Torres Strait Islander Social Survey
OR: Odds ratio
PRISMA: Preferred Reporting Items for Systematic reviews and Meta-Analyses
