# Supplementary material for: Racism in Australia: a protocol for a systematic review and meta-analysis
Source: Syst Rev. 2022 Mar 17;11:47. doi: 10.1186/s13643-022-01919-2 (PMC8929717; doi:10.1186/s13643-022-01919-2)
Supplement: Supplementary file 1 — Additional file 1. Racism in Australia. [file 13643_2022_1919_MOESM1_ESM.docx]

**Additional file 1: Search Strategy**

MEDLINE Search Strategy (adapted for other databases as needed)

Terms:

1. discrim* (ts)
2. prejud* (ts)
3. racis* (ts)
4. Islamophob* (ts)
5. “anti semit*” (ts)
6. stigma* (ts)
7. stereotyp* (ts)
8. racial* (ts)
9. “race related stress” (ts)
10. 1-9 (OR) (ts)
11. Australia* (ts)
12. “cross section*” (ts)
13. quantitative* (ts)
14. survey* (ts)
15. questionnaire* (ts)
16. data (ts)
17. longitudinal* (ts)
18. prevalen* (ts)
19. 12-18 (OR)
20. 10 AND 11 AND 19
